# Comparing the rates of speciation and extinction between phylogenetic trees

**DOI:** 10.1002/ece3.4030

**Published:** 2018-05-04

**Authors:** Liam J. Revell

**Affiliations:** ^1^ Programa de Biología Universidad del Rosario Bogotá Colombia; ^2^ Department of Biology University of Massachusetts Boston Boston MA USA

**Keywords:** comparative methods, diversification, maximum likelihood, phylogeny

## Abstract

Over the past decade or so it has become increasingly popular to use reconstructed evolutionary trees to investigate questions about the rates of speciation and extinction. Although the methodology of this field has grown substantially in its sophistication in recent years, here I will take a step back to present a very simple model that is designed to investigate the relatively straightforward question of whether the tempo of diversification (speciation and extinction) differs between two or more phylogenetic trees, without attempting to attribute a causal basis to this difference. It is a likelihood method, and I demonstrate that it generally shows type I error that is close to the nominal level. I also demonstrate that parameter estimates obtained with this approach are largely unbiased. As this method can be used to compare trees of unknown relationship, it will be particularly well‐suited to problems in which a difference in diversification rate between clades is suspected, but in which these clades are not particularly closely related. As diversification methods can easily take into account an incomplete sampling fraction, but missing lineages are assumed to be missing at random, this method is also appropriate for cases in which we have hypothesized a difference in the process of diversification between two or more focal clades, but in which many unsampled groups separate the few of interest. The method of this study is by no means an attempt to replace more sophisticated models in which, for instance, diversification depends on the state of an observed or unobserved discrete or continuous trait. Rather, my intention is to provide a complementary approach for circumstances in which a simpler hypothesis is warranted and of biological interest.

## 1 INTRODUCTION

Over the past several decades, the field of phylogenetic comparative biology has emerged to assume a leading role in the study of evolutionary change through time (Felsenstein, 1985; Harvey & Pagel, 1991; O'Meara, 2012). Many phylogenetic comparative methods involve combining evolutionary trees with trait data for species to ask questions about how phenotype may have evolved over the history of our group of study (O'Meara, 2012; e.g., Butler & King, 2004; Felsenstein, 1985; Hansen, 1997; Mahler, Revell, Glor, & Losos, 2010; O'Meara, Ané, Sanderson, & Wainwright, 2006; Revell, Mahler, Peres‐Neto, & Redelings, 2012). However, a separate class of method uses phylogenies in which the lengths of the branches in the tree have been calibrated to be equal or proportional to elapsed time. These time‐calibrated phylogenies, or time‐trees, can then be employed to investigate questions about the processes under which lineages accumulate—namely, speciation and extinction (Nee, May, & Harvey, 1994; Stadler, 2012). The first explicitly statistical method designed to estimate speciation and extinction rates from a reconstructed phylogenetic tree was proposed by Sean Nee and colleagues in the first half of the 1990s (Nee et al., 1994; although prior methods based on sister‐group comparisons also existed at this time, e.g., Slowinski & Guyer, 1993). The approach of Nee and colleagues utilizes the internode distances from a fully sampled and time‐calibrated phylogenetic tree. On a semilogarithmic scale, the slope (or rate) of new lineage accumulation in the reconstructed phylogeny has an expected value at the root of the tree equal to the net diversification rate: speciation minus extinction. As the present day is approached the value of this slope will tend toward the speciation rate alone, because extinction has not yet had time to act on these most recent lineages of the tree. This property of reconstructed phylogenies (called the “pull of the present”) is exploited in the method of Nee et al. (1994) to estimate the rates of lineage proliferation and loss from the phylogeny.

The approach of Nee et al. (1994) has subsequently spawned a cottage industry of new, increasingly sophisticated approaches and techniques. Some notable examples include a method developed by Maddison, Midford, and Otto (2007) and Fitzjohn, Maddison, and Otto (2009) designed to measure the association of a binary trait with speciation and extinction rates, and a flexible approach by Rabosky (2014) designed to model continuous heterogeneity in the rates of speciation and extinction on the tree. These methods are not alone, however, and numerous others have been presented within the past decade or so (e.g., Alfaro et al., 2009; Morlon, Potts, & Plotkin, 2010; Goldberg, Lancaster, & Ree, 2011; Silvestro, Schnitzler, & Zizka, 2011; Etienne & Haegaman, 2012; Bealieau & O'Meara, 2016; reviewed in Ricklefs, 2007; Stadler, 2012; Rabosky, Mitchell, & Chang, 2017).

Unfortunately, several of these new approaches have also been criticized. For instance, Rabosky and Goldberg (2015; also see Maddison & Fitzjohn, 2015 and O'Meara & Beaulieu, 2016) showed that in empirical phylogenies, there is a concerning tendency to reject constant rate speciation and extinction in favor of a trait‐dependent model, even if the trait in question has been created with no association with the generative process of the tree. This seems to be because the null model of constant rates is overly simplistic for virtually all empirical phylogenetic trees, and thus any degree of model complexity that can help explain the genuine underlying heterogeneity in diversification rates on the phylogeny is favored (O'Meara & Beaulieu, 2016). More recently, Moore, Höhna, May, Rannala, and Huelsenbeck (2016) published a challenging critique of Rabosky's (2014) Bayesian approach, and it has been argued that it is simply not possible to model speciation and extinction rates drawn from a continuous distribution as in the method of Rabosky (2014; see Höhna et al., 2017; but see Rabosky et al., 2017).

To try to circumvent some of this heated controversy, here I have deliberately taken a step back to present a relatively simple model in which, given a total of *m* trees with branch lengths in matching units of time, we fit one model in which all trees are constrained to diversify under an identical process of speciation and extinction. Then, we compare this model to one in which each tree is allowed to have its own tree‐specific diversification rates. I can and do extend this general approach to test hypotheses in which all trees share a common rate of extinction, but differ in their speciation rates; in which all trees share a speciation rate, but different in extinction; and in which a subset of phylogenies in a group has diversified under a common process that in turn differs from the remaining trees. Any or all phylogenies can have an incomplete sampling fraction, as long as we are comfortable with the assumption that this fraction is known for each tree, and that the missing lineages are absent at random (Stadler, 2012). After briefly describing the model in its various flavors, I will then proceed to examine its statistical properties using numerical simulations. Finally, I will conclude by discussing some potential uses for this method, some common sources of error and bias in the estimation of diversification rates from phylogenies in general, and some alternative models for the processes of speciation and extinction through time.

## 2 MODEL, METHODS, AND RESULTS

### 2.1 The model

The method employed herein is a very simple extension of Nee et al. (1994) and Stadler (2012). In it, we will consider two models. One is a more complex model in which the speciation rates (λ_1_, λ_2_, λ_3_, and so on) and the extinction rates (μ_1_, μ_2_, μ_3_, and so on) of each phylogeny are permitted to assume different values. This model includes known incomplete sampling fractions (denoted ρ) that are permitted to differ from tree to tree. We can compute the log‐likelihood under this model by merely summing the separate log‐likelihoods across our various phylogenies as follows:

$$\begin{array}{cc} \text{log}(L) & =\sum_{i=1}^{m}[(\text{log}\left(\left(N_{i}-1\right)!\right)+2\log\left(p_{1}^{i}\left(t_{1}\right)\right)\\ & -2\text{log}(1-p_{0}^{i}\left(t_{1}\right)))+\sum_{j=2}^{N_{i}-1}\text{log}(\lambda_{i}p_{1}^{i}\left(t_{j}\right))] \end{array}.$$

here, *m* is the total number of reconstructed phylogenetic trees in our study, *N*_i_ is the number of species sampled in the *i*th tree, *t*_*j*_ is the *j*th branching time in distance from the present, ordered from root to tip, while $p_{0}^{i}\left(t\right)$ and $p_{0}^{i}\left(t\right)$ are defined for the *i*th tree as follows (Stadler, 2012):

$${p_{0}}^{i}\left(t\right)=1-\frac{\rho_{i}\left(\lambda_{i}-\mu_{i}\right)}{\rho_{i}+\left(\lambda_{i}\left(1-\rho_{i}\right)-\mu_{i}\right)e^{-\left(\lambda_{i}-\mu_{i}\right)t}}$$

$$p_{0}^{i}\left(t\right)=\frac{\rho_{i}{\left(\lambda_{i}-\mu_{i}\right)}^{2}e^{-\left(\lambda_{i}-\mu_{i}\right)t}}{{\left(\rho_{i}\lambda_{i}+\left(\lambda_{i}\left(1-\rho_{i}\right)-\mu_{i}\right)e^{-\left(\lambda_{i}-\mu_{i}\right)t}\right)}^{2}}.$$

This is exactly the same as model (5) in Stadler (2012), in which we condition on the total depth of the most recent common ancestor of each clade, but here we merely accumulate this likelihood across trees. Note that it would be straightforward to extend this approach to other conditionings, as presented in Stadler (2012), so long as likelihood expressions are available.

Next, we can then compare this to a simpler model in which λ_1_ = λ_2_ = … = λ_*m*_ and in which μ_1_ = μ_2_ = … = *u*_*m*_. The former model has 2*m* parameters for *m* different phylogenies (one speciation and one extinction rate for each tree); whereas the latter has only two parameters to be estimated: the single, global speciation and extinction rates, λ and μ. As such, we can easily compare the likelihoods of the two models using a likelihood‐ratio test with 2*m* − 2 degrees of freedom (or using any of the normal machinery of likelihoods).

In addition to these two models, it is also straightforward to fit a model in which all speciation rates are permitted to assume different values (λ_1_ ≠ λ_2_ ≠ … ≠ λ_*m*_), but extinction rates are equal among trees (μ_1_ = μ_2_ = … = μ_*m*_), or one in which all speciation rates are equal (λ_1_ = λ_2_ = … = λ_*m*_) but extinction rates can differ among phylogenies (μ_1_ ≠ μ_2_ ≠ … ≠ μ_*m*_). Both of these models would have *m* + 1 parameters to be estimated, and thus could be compared to the simplest model in which all rates are equal across trees using a likelihood‐ratio test with *m* − 1 degrees of freedom. Similarly, it would be straightforward to model diversification as a “Yule” or pure speciation process in which μ_1_ = μ_2_ = … = μ_*m*_ = 0. In this case, we could permit all speciation rates to assume different values and compare this to a Yule model with a constant speciation rate across trees using a likelihood‐ratio test with *m* − 1 degrees of freedom. Finally, if we have an a priori hypothesis about variation in the rate of diversification among trees, for instance, that trees 1, 2, and 4 share a common set of speciation and extinction rates (λ_1_ = λ_2_ = λ_4_ and μ_1_ = μ_2_ = *u*_4_), while tree 3 arose via a different process, we can fit this model and compare it to our simplest model in which all rates are constrained to be equal. In this final case, the number of parameters in the more complex of the two models is 2*m* in which *m* is the number of groups, rather than the number of phylogenies; whereas in the simpler model the number of parameters to be estimated is still merely two.

### 2.2 Simulation tests of the method and results

**Figure 1:**
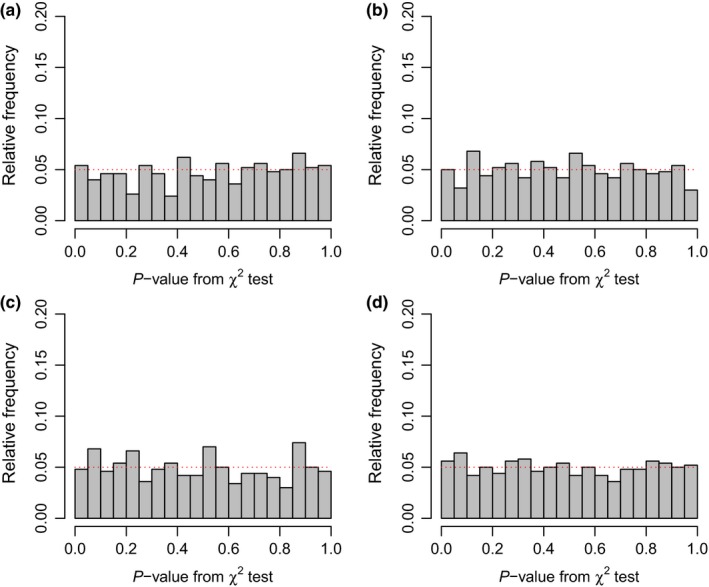
Distribution of *p*‐values obtained from hypothesis tests when data were simulated under the null hypothesis of no difference in rate between trees. The expected distribution is uniform on the interval of [0, 1]. Simulation conditions were selected to result in three phylogenies each with an equal expected number of lineages of 50, 100, 200, and 500 for panels (a) through (d), respectively, given a total tree depth, *T*, of 100, while maintaining a constant extinction fraction μ/λ = 0.25. Specific simulated speciation (λ) and extinction (μ) rates were as follows: (a) λ_1_ = λ_2_ = λ_3_ = 0.043 and μ_1_ = μ_2_ = μ_3_ = 0.011; (b) λ_1_ = λ_2_ = λ_3_ = 0.052 and μ_1_ = μ_2_ = μ_3_ = 0.013; (c) λ_1_ = λ_2_ = λ_3_ = 0.061 and μ_1_ = μ_2_ = μ_3_ = 0.015; and (d) λ_1_ = λ_2_ = λ_3_ = 0.074 and μ_1_ = μ_2_ = μ_3_ = 0.018

To explore the statistical properties of this method, I first conducted a simple analysis of its type I error. For this analysis, I simulated 500 sets of three trees under different speciation and extinction rates. I held the extinction fraction, μ/λ, constant at 0.25, and I varied net diversification (λ − μ) such that the expected number of lineages after *T* = 100 units of time were E(N)=20, 50, 100, 200, or 500, depending on the simulation. I then computed the fraction of analyses resulting in a significant result via a likelihood‐ratio test for each set of simulation conditions. For all of these sets of simulations, the simulated speciation and extinction rates were identical among trees, and thus rejection of the null of common diversification rates in favor of any of the aforementioned alternative hypotheses (variable speciation, variable extinction, or variable speciation & extinction) would represent an error of the first type. In addition to the null model of equal rates, I also fit all three of the aforementioned models to each set of phylogenies. The results from this analysis are presented in Figure 1 (also see Figures S1 and S2) and Table 1 (also see Tables S1 and S2). *p*‐Values across simulations for all simulation conditions described above reasonably approximated a uniform distribution on the interval [0, 1], which is exactly as predicted under the null hypothesis (Figures 1, S1, and S2)—that is, if the statistical method is working as designed. In addition, in exactly half of my simulation conditions, the type I error rate of the method did not significantly exceed the nominal rate of 0.05 based on a binomial test (Tables 1, S1, and S2). In particular, no type I errors using the full (variable speciation and extinction) model were significantly elevated above the nominal level (Table 1); while three of four simulation conditions in each of the variable speciation and variable extinction models significantly exceeded the 0.05 threshold Tables (S1 and (S2). Note that even in cases in which type I error was significantly elevated based on a binomial test, the highest observed type I error rate of this study was less than 10% (specifically, 0.085; Table S2).

**Table 1:** Type I errors for the variable speciation and extinction model with data generated under the null hypothesis of equal speciation and extinction rates between trees. Each analysis consisted in generating three phylogenies with an equal expected number of extant lineages equal to E(N)=50, 100, 200, or 500. Each simulation condition was replicated 500 times

Expected number of lineages, *E*(*N*)	Type I error rate	*P* (binomial test)
50	0.057	0.296
100	0.051	0.447
200	0.049	0.529
500	0.056	0.232

**Figure 2:**
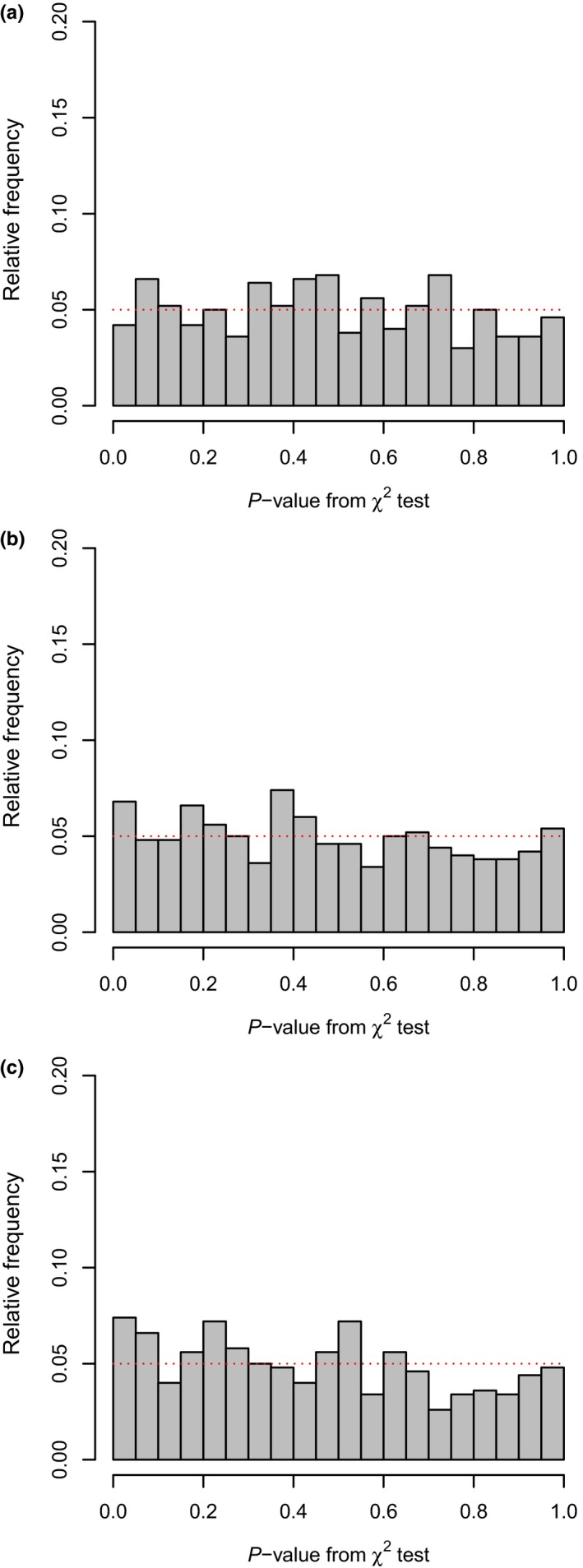
Distribution of *p*‐values obtained from null hypothesis testing in which the data were simulated with no difference in speciation or extinction rates between trees. The expected distribution is uniform on the interval [0, 1]. Speciation and extinction rates for each of two trees generated for each replicate were identical at λ_1_ = λ_2_ = 0.052 and μ_1_ = μ_2_ = 0.013; however, simulations were conducted over different total durations of *T*_1_ = 100 and *T*_2_ = 141.14 to result in a different expected number of lineages in each tree (100 and 500, respectively). Panel (a) shows the distribution of *p*‐values in comparing the variable speciation/variable extinction to the null hypothesis of no difference in speciation or extinction among trees; while panels (b) and (c) show the variable speciation and variable extinction models, respectively

I also simulated under the null hypothesis but in which I varied *T*, the total depth of the tree, such that the expected number of taxa was unequal between the two trees. In particular, I fixed the birth rate, λ, to 0.052, and the death rate, μ, to 0.013, and then simulated two trees each of total depth *T*_1_ = 100 and *T*_2_ = 141.1, respectively. These simulation conditions were chosen because they result in an expected number of lineages equal to $E(N_{1})=100$ and $E(N_{2})=500$ for each phylogeny. I simulated 500 pairs of trees in this way, and fit each of the three aforementioned alternative models (variable speciation, variable extinction, and variable speciation & extinction) along with our null model of equal rates among trees. For all three models, the distribution of *p*‐values across simulated datasets closely resembled a uniform distribution on the interval [0, 1], just as expected for data generated under the null (Figure 2). Type I error was not significantly elevated above its nominal level of 0.05 for the variable speciation & extinction model (Table 2); however, type I error was significantly elevated for both the variable speciation and the variable extinction models (Table 2). The highest observed level of type I error was 0.075 (Table 2).

**Table 2:** Type I errors for the variable speciation & extinction, variable speciation, and variable extinction models for phylogenies generated under the null hypothesis of no difference in speciation or extinction rate between trees. Each of 500 replicated analyses consisted in generating two phylogenies with equal speciation and extinction rates, but differing in total depth such that the expected number of lineages in each tree were $E(N_{1})=100$ and $E(N_{2})=500$, respectively, then fitting each of the three aforementioned models (plus the equal rates null model) to each pair of trees

Model	Type I error rate	*P* (binomial test)
Variable speciation & extinction	0.042	0.759
Variable speciation	0.069	0.030
Variable extinction	0.075	0.008

### 2.3 Power and parameter estimation

**Figure 3:**
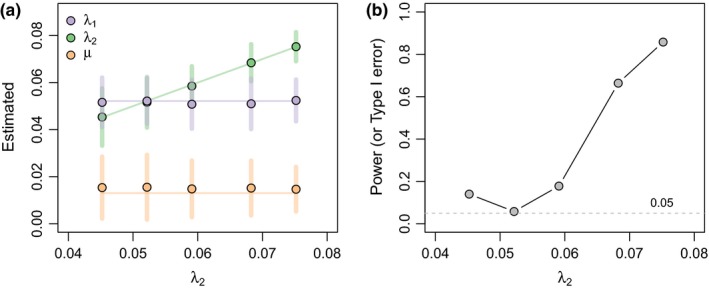
Parameter estimation and power of the variable speciation method for data simulated under a scenario in which the speciation rate, but not the extinction rate, differed between trees. Simulation conditions were selected to result in an expected number of extant lineages of 100 in tree 1 and 50, 100, 200, 500, or 1,000 in tree 2, while maintaining a constant extinction rate of μ = 0.013 and a constant total tree depth of *T* = 100. Panel (a) shows the mean parameter estimate compared to the generating values for λ_1_, λ_2_, and μ. Horizontal or diagonal lines represent the generating values of the simulations, and vertical bars show the standard deviation of the estimated values across simulations. Panel (b) gives the power (or type I error, in the case of no difference in speciation rate between trees) of the method

In addition to this analysis of type I error, I also examined power and parameter estimation of the method when the null hypothesis of equal speciation and extinction among trees was false. To do this, I simulated 500 pairs of trees under each of the following simulation conditions: I held total depth constant at *T* = 100, I held extinction constant at μ = 0.013, and I set λ_1_ = 0.052. I then varied λ_2_ from λ_2_ = 0.045, 0.052, 0.059, 0.068, to 0.075. These parameter values were selected because they result in $E(N_{1})=100$ and $E(N_{2})=50$, 100, 200, 500, and 1,000, respectively. For all parameter values, I repeated any simulation that resulted in fewer than five extant lineages in the reconstructed phylogeny. For each pair of simulated trees, I fit a variable speciation model (the known generating model) and recorded the parameter estimates and *p*‐value of the null hypothesis test against a model of equal speciation and extinction rates among trees. Figure 3a shows that parameter estimates were unbiased for both λ_1_ and λ_2_. The mean, common extinction rate is slightly biased in an upwards direction, although this is likely due to the fact that estimation of the rate of extinction, by convention (and logically), has a lower bound of μ = 0.0. Figure 3b shows that power to reject the null hypothesis of equal rates was relatively modest when differences in the generating speciation rates (λ_1_ and λ_2_) are small; however, power increases to nearly 0.86 for the largest difference in rate simulated in this study. I also conducted precisely the converse analysis in which I held speciation rate constant and varied extinction rate, once again using parameter values chosen to result in $E(N_{1})=100$ and $E(N_{2})=50$, 100, 200, 500, and 1,000. I then evaluated parameter estimation and power of the method. The results from this analysis are similar and shown in Figure S3. Finally, I conducted an analysis in which both speciation and extinction rate were varied between trees. I selected parameter values for λ_1_, λ_2_, μ _1_, and μ_1_ such that $E(N_{1})=50$, 100, 200, 500, and 1,000, while $E(N_{2})=1,000$, 500, 200, 100, and 50, using intersecting extinction fractions (μ/λ) from 0.35 to 0.15 and 0.15 to 0.35, respectively. The results from this analysis are given in Figure S4.

Although the decision to retain only trees with greater than four tips has the potential to slightly bias the results (by removing trees that experienced either a very low number of speciations or an unusually high number of extinctions for a given set of μ and λ), I have no reason to suspect that this should increase measured power to detect a significant difference in diversification between trees. To the contrary, by removing “extreme phylogenies” (that is, trees with far fewer taxa than expected) one instead might predict measured power of the method to be decreased relative to its true value when a genuine difference in rate has been simulated. Furthermore, when I duplicated this analysis using all trees containing greater than two taxa instead of only trees with greater than four taxa (not shown) the power of the method and average results were unaffected (although the variance estimates among simulations increased substantially for the lowest net diversification rate simulations).

In addition to these analyses, I also conducted several additional tests of the method performance with regard to number of trees and total tree size. First, I simulated birth–death trees in which tree 1 was generated using a speciation rate (λ = 0.067) expected to result in $E(N_{1})=300$ after 100 units of time, while trees 2 to *n* for *n* = 2 through 11 were simulated using a speciation rate expected to result in 100 taxa after the same amount of time (λ = 0.056), holding extinction rate constant at μ = 0.017. I then fit both variable speciation and constant speciation & extinction models to each simulated set of trees. I found that power to reject the null hypothesis of constant speciation among phylogenies did not seem to vary with the total number of trees and was around 40% for all simulation conditions (Figure S5). To examine the statistical power of the method for trees containing very few taxa, I also simulated pairs of pure‐birth (“Yule”) trees in which I held speciation rates constant at λ_1_ = 0.04 and λ_2_ = 0.08 for all simulations, but in which I varied *T*_1_ and *T*_2_ such that the expected number of lineages (*N*_1_ and *N*_2_) were identical for all simulation conditions and equal to 5, 10, 20, 40, and 80. In contrast to all prior simulations, I also conditioned simultaneously on λ, μ (set to μ = 0 for all simulations), *N*, and *T*. This is more computationally intensive, but results in trees for each simulation condition that are invariant in taxon number and total depth. I then fit a both variable speciation and constant speciation Yule models and measured statistical power as the frequency of tests in which the null hypothesis of constant speciation was rejected. The results from this analysis showed very low power for the smallest phylogenies, but statistical power in excess of 50% even for trees simulated with *N*_1_ = *N*_2_ = 20, given the simulated difference in birth rate (Figure S6).

### 2.4 Notes on implementation

All the models and methods of this study have been implemented for the R statistical computing environment (R Core Team, 2017), and all simulations and analyses were conducted in R. The statistical method described herein has been implemented as an option of the *ratebytree* function of my *phytools* R package (Revell, 2012). *phytools* itself in turn depends on the important R phylogenetics packages *ape* (Paradis, Claude, & Strimmer, 2004) and *phangorn* (Schliep, 2011), as well as on a number of other R packages (Azzalini & Genz, 2016; Becker, Wilks, Brownrigg, Minka, & Deckmyn, 2016; Chasalow, 2012; Gilbert & Varadhan, 2016; Harmon, Weir, Brock, Glor, & Challenger, 2008; Jackson, 2011; Lemon, 2006; Ligges & Mächler, 2003; Neuwirth, 2014; Pinheiro, Bates, DebRoy, Sarkar, & R Core Team, 2017; Plummer, Best, Cowles, & Vines, 2006; Qiu & Joe, 2015; Venables & Ripley, 2002; Xie, 2013).

## 3 DISCUSSION

### 3.1 Type I error

This method had type I error rates close to the nominal level under a range of conditions (Figures 1 and 2; Tables 1 and 2). In fact, in many simulations, type I error rates were not significantly different from 0.05 based on a binomial test (e.g., Table 1). However, in about half of all simulations type I error was significantly elevated, though the highest level of type I error observed across all simulations was 0.085 (Tables S1 and S2). In empirical research, I recommend accompanying a likelihood‐ratio test with a test involving a null distribution for the likelihood‐ratio test statistic generated via simulation. Prior research suggests that this is most likely to be important when some parameter or parameters of the model, for instance, the extinction rates, are at or near their boundary condition (e.g., Etienne, Pigot, & Phillimore, 2016). This is already straightforward to undertake in the R environment given the abundant range of phylogenetic simulators available in R (e.g., Harmon et al., 2008; Paradis et al., 2004; Revell, 2012; Stadler, 2017). Herein, I did not conduct a thorough exploration of this approach, given the computational intensity of the requisite simulations and the relatively good statistical performance of the method when using a simple χ^2^ distribution as the null. In addition, generating the null distribution via simulation opens a number of questions that I am relatively unprepared to answer. In particular, it is not entirely clear to me whether simulated trees should be conditioned on total depth, λ, and μ, or on depth, λ, μ, and the total number of taxa in each observed tree (Stadler, 2011). This is an interesting question that could be the subject of future study; however, in the meantime, I recommend treating statistically marginal results with caution.

### 3.2 Use of the method

**Figure 4:**
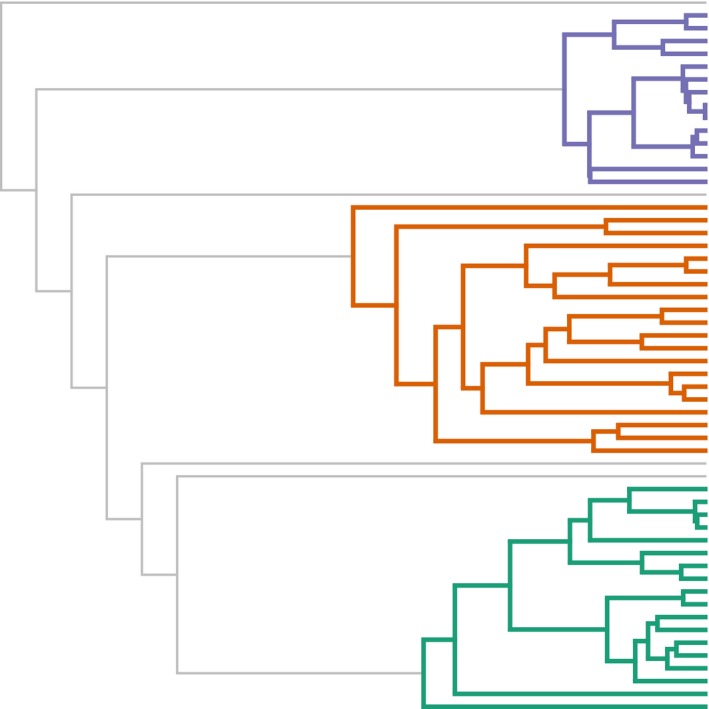
Example scenario for this method for comparing diversification rates among trees, but in which the relationship among phylogenies is known or hypothesized. The various colored subtrees represent the clades of interest and of known sampling fraction, where the remaining gray branches show poorly sampled lineages or lineages of unknown sampling fraction, and for which no a priori hypothesis exists for variation in speciation or extinction rates across the phylogeny

As noted in the introduction, recent years have witnessed the rapid proliferation of methodology designed to investigate heterogeneity in the process of diversification throughout the tree of life. Some of these methodologies have been criticized (e.g., Moore et al., 2016; Rabosky & Goldberg, 2015); nonetheless, I feel that these approaches continue to remain state‐of‐the‐art for the field. Herein, I have presented a simpler technique for exploring heterogeneity in the process of diversification among phylogenies. In it, I proposed merely accumulating the likelihood across trees under a scenario in which all trees share a common set of speciation and extinction rates, and then under another scenario in which each tree is permitted to have its own unique set of rates. Then, we just need to compare the likelihoods. Although I envision applying this approach to phylogenetic trees of unknown relationship—an equally common situation might be the one illustrated by Figure 4 in which some parts of the tree are of interest (in this case represented by the variously colored subtrees of the figure), have been well‐sampled and are of known sampling fraction; whereas the remainder of the tree is of less interest and is poorly sampled or of unknown sampling fraction. In this case, we can merely extract the various subtrees of interest and fit both a model in which they share common rates, and one in which rates are permitted to differ between subtrees of the phylogeny and compare them.

Note that in comparing this simple method to other more sophisticated (if recently controversial) approaches, I in no way intend to imply that I have proposed a replacement for these. Instead, I merely mention the comparison because in some cases in which these methods have proven inappropriate, the approach presented herein may nonetheless be useful. For instance, if we imagine a scenario in which clades A and B arose under a low rate of diversification, whereas clade C was produced by a different higher rate, a significant model fit via a state‐dependent diversification method, such as the BiSSE method of Fitzjohn et al. (2009) using a character that happened to be fortuitously associated with the taxa of clade C would be weak evidence at best of a genuine causal association between our trait and elevated diversification. In fact, absent data from other groups in addition to our three aforementioned clades, no method can genuinely prove a causal link between our trait and elevated diversification in clade C. On the other hand, it would be completely reasonable to ask if clades A and B diversified by one process and C by another, or if all three had arisen under similar speciation and extinction rates (and perhaps differ one from the other in species richness due to chance alone). This simpler question can be asked and answered using the methodology of this paper, just so long as we understand that a significant result in no way implies a causal link between diversification and any trait.

Finally, it is also worth mentioning that for cases in which one or more changes in rate are suspected, but no specific a priori hypothesis for the positions of these shifts is available, I would not recommend employing the approach of this study to test every conceivable hypothesis for rate variation among clades. Instead, for the situation of a single tree one might employ the hidden rates model of Bealieau and O'Meara (2016). For multiple trees, it is straightforward to envision designing a reasonably straightforward reversible‐jump Markov Chain Monte Carlo (MCMC) approach using the likelihood expressions of this article. In this case, Bayesian MCMC would be used to sample models of varying complexity and parameter values from their joint posterior distribution. Although an intriguing idea, I consider this to be beyond the scope of the current study.

### 3.3 Biases and sources of error in the estimation of diversification rates

The birth–death model that is used to approximate the accumulation of diversity in phylogenetic trees in this study, and throughout the literature, is a stochastic process with high variance. Consequently, it will often be quite difficult to accurately determine the generating parameters of this process from empirical trees, even under the idealized circumstances of phylogenetic trees estimated with no or minimal error. Nonetheless, there are also attributes of the statistical methods used to estimate trees, and the nature of the phylogenetic datasets typical of contemporary phylogenetic studies, that can contribute bias to the estimation of speciation and extinction rates from reconstructed trees.

For instance, it has been shown that model insufficiency (e.g., Revell, Harmon, & Glor, 2005) will cause the deepest edges of the tree to be systematically underestimated relative to edges closer to the present day. This may cause the perception of a “slowdown” in the accumulation of new lineages toward the present, counterbalancing the “pull‐of‐the‐present” that is used by the method of Nee et al. (1994; and thus too by this method) to measure extinction. Consequently, underestimation of deep edges in the tree should systematically downwardly bias the estimation of extinction rates from empirical molecular phylogenies.

Similarly, most contemporary phylogenetic studies use molecular genetic data to infer the relationships of species. These data often consist of gene sequences from many loci. Population genetic coalescence invariably precedes speciation events. In fact, theory predicts that coalescence should precede speciation by (on average) 2*N*_*e*_ generations, in which *N*_*e*_ is the effective population size of the parental lineage before the event. This is not ameliorated by including data from multiple loci. In fact, so doing merely helps guarantee (for more and more independently segregating loci) that the average divergence time precisely matches the divergence time predicted by coalescent theory: that is, the true time of speciation minus 2*N*_*e*_ generations. If effective population size and generation times are relatively constant across the tree then all internal nodes of the tree should be affected equally—pushed an equal distance backwards in time relative to the true times to speciation. Terminal nodes, however, are not so affected and will have an expected length equal to the sum of their true length plus the time to coalescence. Consequently, coalescence may also have the effect of increasing the length of terminal edges relatively to internal edges of the tree, and thus should downwardly bias the estimation of extinction rates from molecular phylogenies. This will be particularly true of rapid diversifications in which edge lengths and coalescent times are similar in magnitude.

Finally, contemporary diversification methods, including the simple approach of this article, can now readily take into account incomplete sampling fraction (Stadler, 2012); however, these methods invariably assume that the missing taxa are absent from the tree at random. Of course, in empirical studies this is seldom if ever the case. More often, due to prevailing taxonomic practices, I suspect that missing lineages will be overdispersed. Overdispersed missing taxa will disproportionately affect recent nodes of the tree which will also tend to weaken our measured “pull‐of‐the‐present” and thus result in systematically underestimated extinction. (Although it also occurred to me that missing taxa may sometimes be clumped—for instance, if some geographic regions are more poorly studied than others and if species within a region tend to be more closely related than expected by chance. In fact, I do not know how clumped missing taxa would affect the estimation of speciation and extinction by this or other methods.) Inasmuch as some of these sources of bias in the estimation of diversification rate vary among trees, they should also be considered when applying this approach for studying heterogeneity in speciation and extinction between phylogenies.

### 3.4 Some consideration of the birth–death model

In this study, I have developed a method for comparing diversification between trees in which I have assumed a model for diversification generally referred to as the “birth–death” model. This model is one in which speciation events (“births”) and extinctions (“deaths”) occur randomly and instantaneously with a given set of rates, λ and μ, even if these rates are permitted to vary among lineages, through time, or, as in this study, between trees. Although this model seems reasonably logical (as an approximation of the true underlying biological process, of course), a number of alternative, conceptually distinct models have also been proposed. For instance, Morlon (2014) outlined a set of 13 distinct models that might be used to study diversification in phylogenies. Some are variants of the birth–death model employed here, such as the character‐dependent diversification models in which birth and/or death rates vary as a function of a trait (e.g., Fitzjohn, 2012; Goldberg et al., 2011; Maddison et al., 2007). Others, such as the “age dependence model” (in which lineages might be more or less likely to speciate or go extinct as they age; e.g., Mooers, Harmon, Wong, & Heard, 2007), the “protracted speciation model” (in which speciation takes time rather than occurring instantaneously; e.g., Purvis, Orme, Toomey, & Pearson, 2009; Etienne & Rosindell, 2012), or the diversity‐dependent diversification model (e.g., Etienne et al., 2016; Rabosky & Lovette, 2008) represent fundamentally different visions for how speciation and extinction occur through time. To the extent that expressions for the likelihood exist for these different models, it would be relatively straightforward to extend the approach of this study (merely involving the accumulation of likelihoods across trees) to alternative, completely different models for diversification through time.

On the other hand, it is also worth pointing out that the method presented herein is also likely to be sensitive to violations in its assumptions. That is, if the process responsible for producing our observed trees differs markedly from that which we have modeled (the birth–death model in this study, or any of those mentioned above), then, we may end up obtaining a misleading result. For instance, if the true process is density‐dependent speciation, and our two or more trees are in different stages of diversity accumulation, modeling branching times under a birth–death process could conceivably lead us to incorrectly conclude that our trees were growing via different processes (different speciation or extinction rates or both) rather than by the same process at different stages of maturity. This vulnerability to model assumption violations is a property of all model‐based statistical methods and not a peculiarity of the approach presented in this study; however, it should nonetheless be kept in mind.

## 4 CONCLUSION

The quantitative study of speciation and extinction using reconstructed phylogenetic trees, pioneered by Nee et al. (1994) over two decades ago, is more popular now than ever. Unfortunately, some recent methods—for instance a commonly‐used approach designed to link diversification to phenotypic trait evolution—have been subject to criticism. Herein, I have proposed a simpler approach for modeling heterogeneity in the rates of speciation and/or extinction among phylogenetic trees, without purporting to attribute a causal basis to this rate heterogeneity. I show that the method has reasonable statistical properties: type I error at or near the nominal level; and parameter estimation that is reasonably if not entirely unbiased. I hope that this method will be useful for situations in which a difference in diversification rate is hypothesized between two or more clades of unknown relationship, or for the equally common scenario in which the relationship between our clades of interest has been estimated, but intervening lineages have low or unknown sampling fraction. I feel that the method of this article is most properly viewed as a simplification of existing approaches, although it is one that I believe will nonetheless be of substantial utility to the rapidly growing community of macroevolutionary biologists presently investigating heterogeneity in the processes of speciation and extinction on phylogenetic trees.

## CONFLICT OF INTEREST

None declared.

## AUTHOR CONTRIBUTIONS

LJR conceived of the project, undertook all aspects of its implementation, and wrote the manuscript.

## DATA ACCESSIBILITY

Data are available on the Dryad Digital Repository (https://doi.org/10.5061/dryad.f8c9165).
