# Supplementary material for: Comparing the rates of speciation and extinction between phylogenetic trees
Source: Ecol Evol. 2018 May 4;8(11):5303–12. doi: 10.1002/ece3.4030 (PMC6010886; doi:10.1002/ece3.4030)
Supplement: Supplementary file 1 [file ECE3-8-5303-s001.pdf]

Supplementary appendix to:

Comparing the rates of speciation and extinction between phylogenetic trees

Liam J. Revell[1,2]

[1]Programa de Biología, Universidad del Rosario, Bogotá, Colombia

[2]Department of Biology, University of Massachusetts Boston, Boston Massachusetts, U.S.A.

Correspondence: Liam J. Revell, Programa de Biología, Universidad del Rosario, Bogotá, Colombia, Email: liam.revell@phytools.org

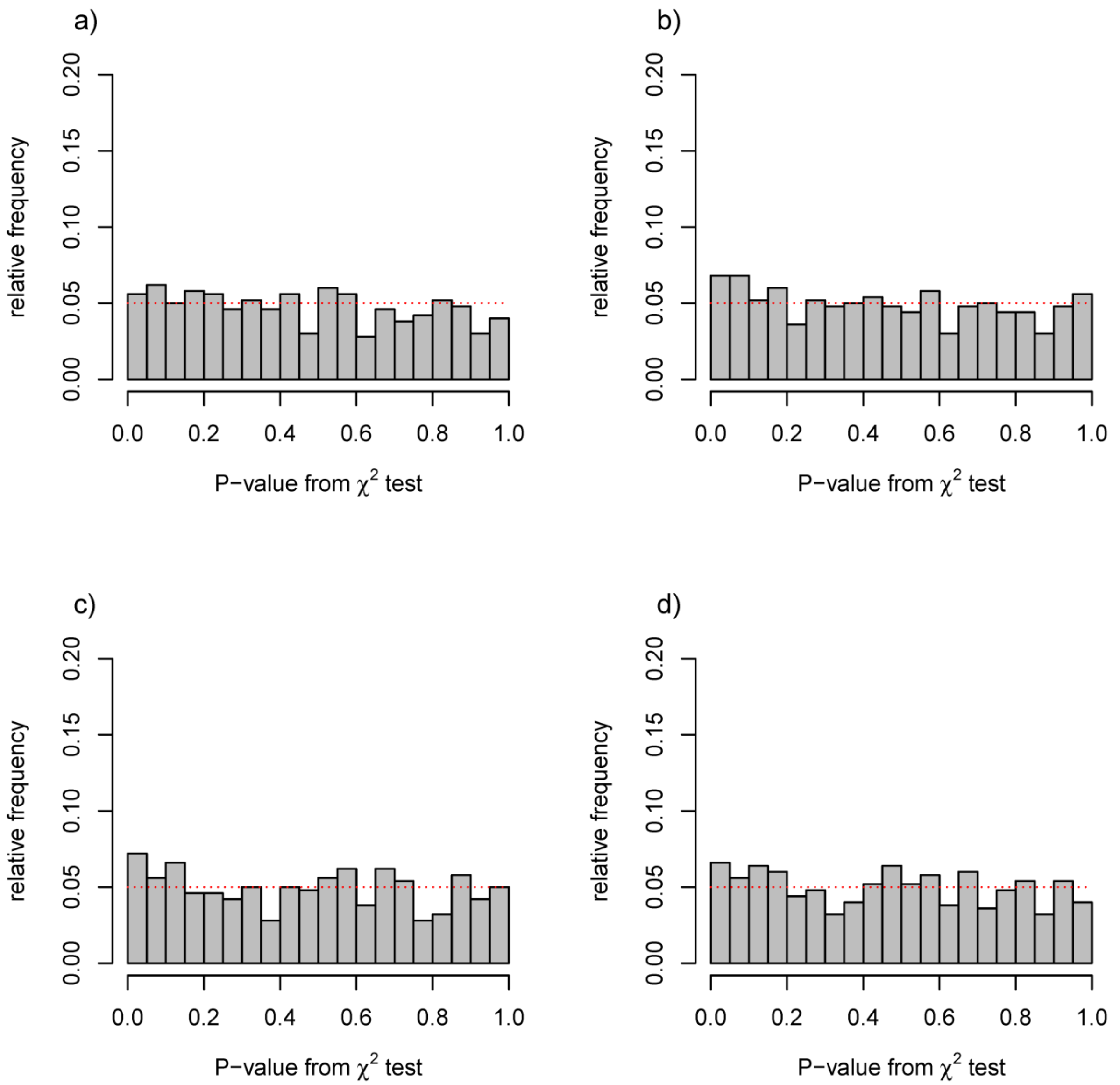

Figure S1. P-values from variable-speciation model for data generated under the null hypothesis of equal speciation and extinction under different circumstances. Simulation conditions are as in Figure 1 of the main text.

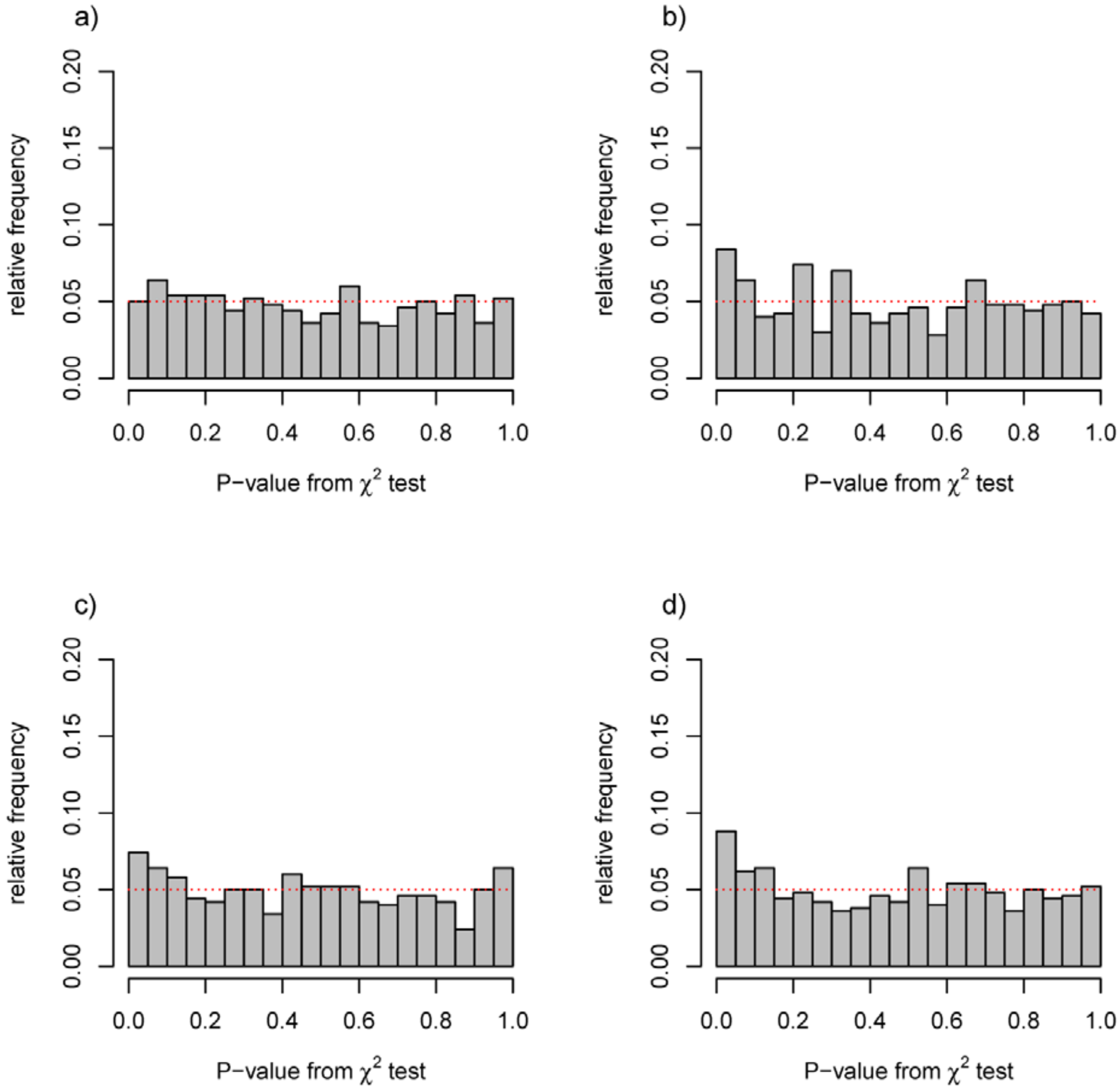

Figure S2. P-values from the variable-extinction model for datasets generated under the null hypothesis of equal speciation and extinction rates under different conditions. Simulation conditions are as in Figure 1 of the main text.

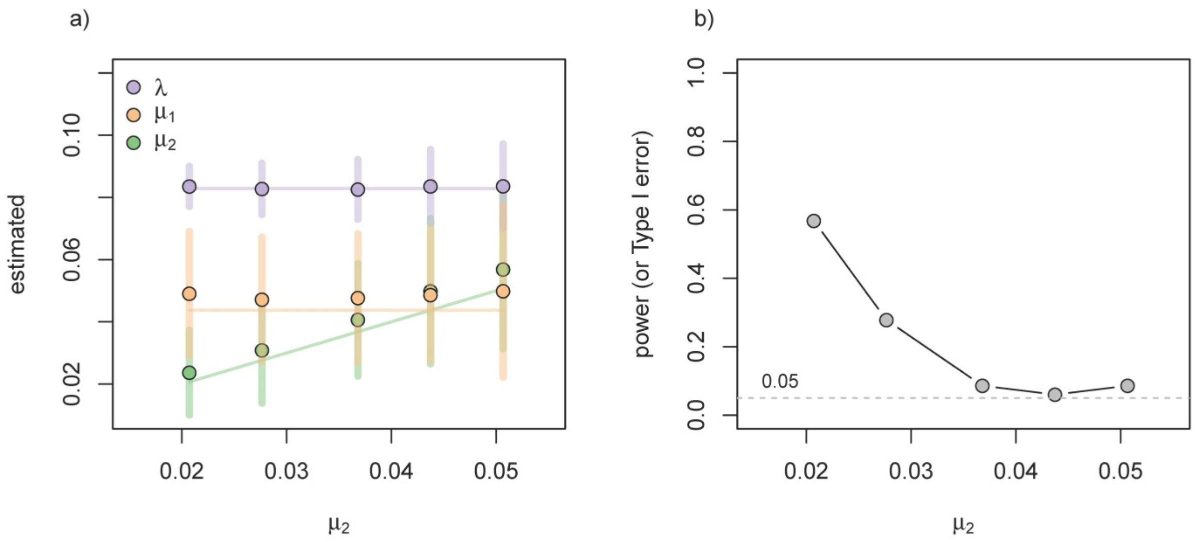

Figure S3. Parameter estimation and power of the variable-extinction method for data simulated under a scenario in which the extinction rate varied among trees, while the speciation rate was held constant. Simulation conditions were selected to result in an expected number of extant lineages of 100 in tree 1 and 50, 100, 200, 500, or 1000 in tree 2, while maintaining a constant speciation rate of $\lambda = 0.08$ and a constant total tree depth of $T = 100$. Panel a) shows the mean parameter estimate compared to the generating values for λ, μ_1, and μ_1. Horizontal or diagonal lines show the generating values, and vertical bars show the standard deviation of the estimated values across simulations. Panel b) gives the power (or type I error in the case of no difference in extinction rate between trees) of the method.

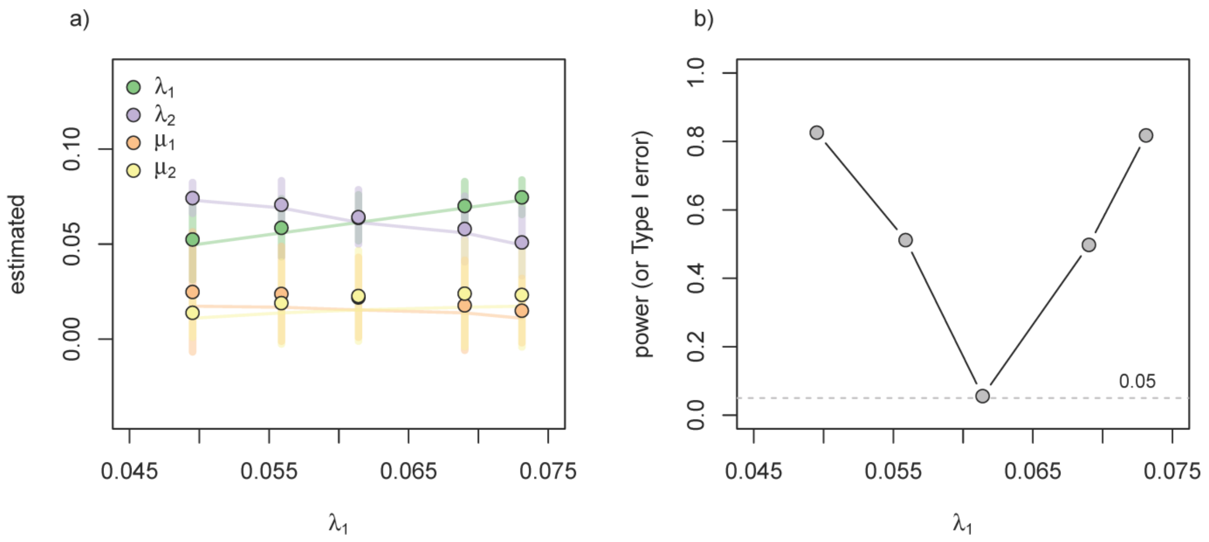

Figure S4. Parameter estimation and power of the variable speciation and extinction method for data simulated under a scenario in which both speciation and extinction rates varied among trees. Simulation conditions were selected to result in an expected number of extant lineages of 50, 100, 200, 500, and 1000 in tree 1 and 1000, 500, 200, 100, and 50 in tree 2, whilst varying the extinction fraction from 0.35 through 0.15 or from 0.15 through 0.35 in intervals of 0.05 or -0.05 for trees 1 and 2, respectively. Consequently, the generating parameters for trees 1 and 2 match precisely when $E(N_1) = E(N_2) = 200$. As in Figures 3 and A3, total tree depth was maintained constant at $T = 100$ for all simulations. Panel a) shows the mean parameter estimate compared to the generating values for λ_1, λ_2, μ_1, and μ_1, show here (arbitrarily) as a function of the value of λ_1 used in each simulation. Plotted lines give the generating values, and vertical bars show the standard deviation of the estimated values across simulations. Panel b) gives the power (or type I error in the case of no difference in speciation or extinction rate between trees) of the method.

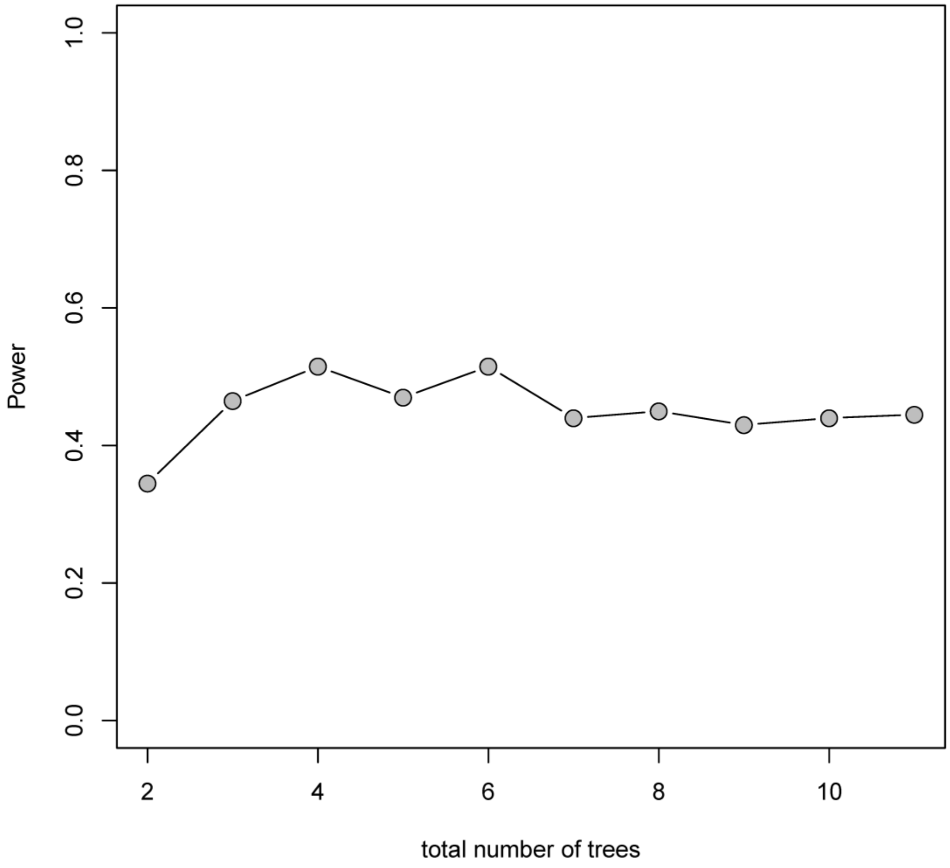

Figure S5. Power of a statistical test for a difference in diversification rate between trees. In all simulation conditions, conducted with a constant death rate of $\mu = 0.017$, tree 1 was simulated with a birth rate expected to result in 300 taxa after 100 units of time ($\lambda = 0.067$), while tree 2 through n for $n = 2$ through 11 was simulated with a birth rate expected to result in 100 taxa after the same amount of time ($\lambda = 0.056$). I then fit a variable-speciation model in which each tree was permitted to have a different speciation rate. 200 simulations were conducted for each condition.

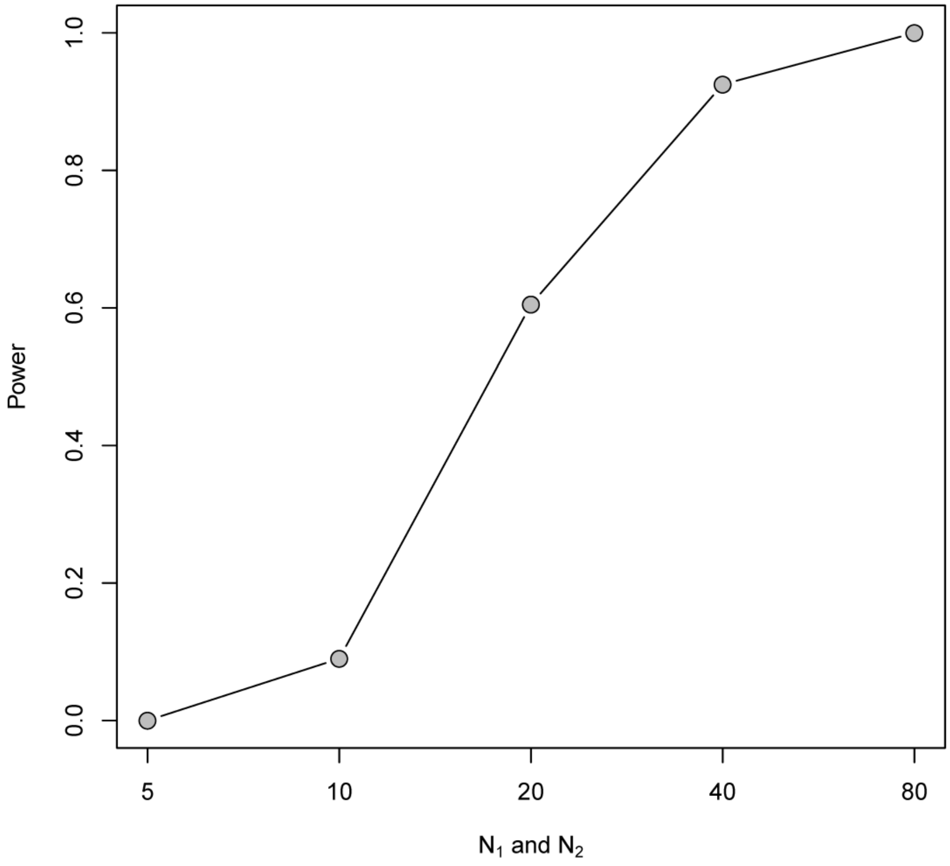

Figure S6. Power of a statistical test for a difference in diversification rate between trees. In each set of simulation conditions, I generated 200 sets of two phylogenies, holding $\lambda_1 = 0.04$ and $\lambda_2 = 0.08$ across all simulations, but varying T_1 and T_2 such that the expected number of lineages (N_1 and N_2) were identical for all simulation conditions. Finally, I sampled phylogenies conditioning simultaneously on λ, μ (set to $\mu = 0$ for all simulations), *N*, and *t*. Results show that power is very low for phylogenies containing 5 and 10 taxa, but for even phylogenies with $N_1 = N_2 = 20$ taxa, power climbs above 50%.

Table S1. Type I errors for the variable-speciation model with data generated under the null hypothesis of equal speciation and extinction rates between trees. Simulation conditions are as in Figure 1 and Table 1 of the main text.

Expected number of lineages, $E(N)$	Type I error rate	P (binomial test)
50	0.059	0.232
100	0.069	0.030
200	0.073	0.012
500	0.066	0.045

Table S2. Type I errors for the variable-extinction model with data generated under the null hypothesis of equal speciation and extinction rates between trees. Simulation conditions are as in Figure 1 and Table 1 of the main text.

Expected number of lineages, $E(N)$	Type I error rate	P (binomial test)
50	0.052	0.447
100	0.085	<0.001
200	0.075	0.008
500	0.088	<0.001
